# Mechanistic insights into how gut homeostasis and immune-system crosstalk shape ankylosing spondylitis

**DOI:** 10.3389/fimmu.2026.1809165

**Published:** 2026-06-08

**Authors:** Jiawen Li, Xingwen Xie

**Affiliations:** Gansu University of Traditional Chinese Medicine, Lanzhou, China

**Keywords:** ankylosing spondylitis, gut – joint axis, gut microbiota, intestinal barrier dysfunction, microbial metabolites, mucosal immunity

## Abstract

Ankylosing spondylitis (AS) is a chronic immune-mediated inflammatory disease that primarily affects the axial skeleton and entheseal structures. Although the role of HLA-B27 is well established, AS pathogenesis is multifactorial, and accumulating evidence suggests that disruption of intestinal homeostasis and the gut–joint axis may contribute to disease pathobiology. AS-associated gut dysbiosis is characterized by reduced microbial diversity and compositional alterations that may be associated with mucosal immune activation, increased intestinal permeability, and systemic inflammatory priming. Mechanistically, altered microbial signals and barrier dysfunction may converge on key immunological pathways, including the IL-23/IL-17 axis, and may promote the activation or trafficking of innate-like lymphocytes, such as MAIT cells, γδ T cells, and ILC3s, thereby contributing to inflammation and abnormal bone remodeling. In addition to community structure, microbial metabolites, including short-chain fatty acids and tryptophan-derived indole metabolites, help regulate epithelial integrity and immunoregulatory homeostasis; their perturbation may favor pro-inflammatory immune programs relevant to AS. This review summarizes recent evidence on dysbiosis, barrier dysfunction, and immunometabolic signaling in the gut–joint axis, while critically distinguishing established immune-targeted therapies from experimental microbiota-directed and combination strategies.

## 1 Introduction

Ankylosing spondylitis (AS) is a persistent inflammatory disease, which is mainly immune in nature and the chief area of its activity is the axial skeleton and entheseal areas (1). Although HLA-B27 role in AS is well-established, the pathogenesis of the disease is much more multifactorial, as it is caused by genetic predisposition factors, bio-immunopathological dysfunction, and environmental factors (2). Recent studies have increasingly implicated intestinal homeostasis in the onset and progression of AS, particularly through the gut–joint axis (3). Changes in gut microbiota, commonly referred to as dysbiosis, have been observed in patients with AS and may provide potential therapeutic opportunities (4). Histological evidence indicates that intestinal inflammation can occur in AS, and a subset of patients may subsequently develop clinically overt inflammatory bowel disease (IBD) (5). The gut–skeletal or gut–joint axis may help explain part of AS pathogenesis, but translating mechanistic evidence into effective cytokine-targeted or microbiome-directed therapies remains challenging (6). The IL-23/IL-17 signaling pathway has been strongly implicated in AS pathogenesis (7). This review synthesizes evidence from 2020–2025 clinical studies, animal models, and mechanistic investigations on how gut microbiota alterations, microbial metabolites, and intestinal barrier dysfunction may influence immune processes relevant to AS (2). Particular attention is given to microbial community dynamics, microbial metabolites, host genetic interactions, intestinal permeability, and mucosal immune activation in AS pathobiology (8). These observations extend current concepts of AS pathogenesis and raise the possibility, not yet fully established, that gut-directed interventions may complement immune-targeted treatment strategies (2).

## 2 The role of gut microbiota dysbiosis in the pathogenesis of ankylosing spondylitis

### 2.1 Microbiome signatures in AS: diversity and composition shifts

A mounting body of evidence suggests that microbial dysbiosis is associated with AS onset and progression, including reduced microbial diversity and compositional shifts characterized in some studies by reductions in Firmicutes and Actinobacteria and increases in Proteobacteria or Enterobacteriaceae (9). Studies using 16S rRNA sequencing have frequently reported reduced α-diversity and compositional changes, although the direction and magnitude of specific taxonomic shifts vary across cohorts (4). Several microbial taxa and functional pathways have been associated with AS disease activity, including altered tryptophan metabolism and microbial enzymes in axSpA patients (10), as well as links between gut dysbiosis and IL-23/IL-17 pathway modulation in AS pathogenesis (11). Although there is some variability among studies and some of the taxa have inconsistent trends, the particular study on IL-17 inhibition in patients with psoriatic arthritis and spondyloarthritis found that specific taxa, including Clostridiales and Candida albicans, change with classifying study patients, and no overall pattern of pro-inflammatory taxa increase and anti-inflammatory microbes reduction was determined (12). These microbial disturbances may reflect inflammation, treatment exposure, host genetics, or diet; nevertheless, they are also hypothesized to contribute to disease initiation or persistence (13–15).

### 2.2 Microbial drivers: antigens and metabolic pathways

AS cohorts show disease-associated mucosal and fecal microbial patterns, although these signatures remain heterogeneous across studies. Fecal samples from AS patients have been reported to show reduced bacterial diversity, decreased Clostridium leptum, and increased Escherichia coli, although the functional significance of these changes remains incompletely defined (16) Prevotella copri and Parabacteroides distasonis have been reported to be enriched in some AS cohorts, but their strain-level functions and causal relevance require further validation (17). Although Prevotella copri has been implicated in AS pathogenesis, current evidence remains insufficient to define the pathogenic role of specific microbial strains, including P. copri. Klebsiella pneumoniae has long been associated with AS, mainly through serological and antigenic-reactivity observations, but current evidence does not establish it as a universal causal pathogen (18). Proposed mechanisms linking gut microbiota and microbial products with AS include interactions with HLA-B27-related host genetics, altered intestinal permeability, and mucosal immune activation (19). Therefore, rather than assigning causality to a single organism, current evidence supports a model in which microbial antigens may contribute to autoimmunity in selected genetic or immunological contexts (2).

### 2.3 Model systems supporting a gut–joint link

Animal models provide supportive, but not definitive, evidence for a gut–joint connection. For example, HLA-B27 transgenic rats can develop both intestinal and joint inflammation under permissive microbial conditions. Studies have demonstrated gut microbiota dysbiosis in spondyloarthritis patients, with genetic background and disease activity influencing microbial composition (20). Experimental models such as curdlan-induced SpA mice have been used to study the impact of microbial-derived butyrate on disease progression and gut microbiota alterations in axial spondyloarthritis (21). Together, these findings suggest that gut microbial alterations may be linked to subclinical intestinal inflammation and innate immune responses, although causality remains to be established (22).

## 3 Impaired intestinal barrier function and initiation of mucosal immune responses

### 3.1 Intestinal barrier dysfunction in as: leaky gut and systemic immune activation

Persistent alterations in the intestinal epithelial integrity, also known as leaky gut, have been implicated in autoimmune diseases, and intestinal barrier dysfunction may contribute to systemic immune activation, although specific evidence for its frequent occurrence in ankylosing spondylitis (AS) remains to be fully established (23). Dysbiosis may contribute to disease-relevant pathways by influencing intestinal barrier function, microbial metabolites, and IL-23/IL-17 signaling, but the directionality of these relationships remains uncertain (2).

### 3.2 Microbial sensing at the mucosa: MAMP–PRR signaling and IL-23 induction

Microbial-associated molecular patterns (MAMPs) can interact with pattern-recognition receptors on innate immune cells, leading to activation of inflammatory signaling pathways and production of pro-inflammatory cytokines, including IL-23 (24), which are implicated in autoimmune pathogenesis.

In addition to TLRs, intracellular NOD-like receptors, particularly NLRP3, are activated in AS, contributing to the maturation of IL-1β and IL-18 through caspase-1 activation (25). Increased expression of NLRP3 has been observed in both clinical samples and animal models, with a strong correlation to disease severity (25). These findings suggest that NLRP3 may represent a potential therapeutic target, although its clinical relevance in AS requires further validation. Indeed, preclinical studies have shown that pharmacological inhibition of NLRP3 can effectively reduce enthesitis and delay the onset of disease.

### 3.3 Mucosal immune circuitry: DC–Th17 differentiation and IL-17–mediated inflammation

Persistent barrier dysfunction may sustain mucosal immune activation in a subset of AS patients. Alterations in immune cell populations, including dendritic cells, have been implicated in ankylosing spondylitis (AS) pathogenesis, with IL-23 playing a role in promoting Th17 cell differentiation. Th17 cells produce IL-17 and other pro-inflammatory cytokines that contribute to inflammation and may affect epithelial integrity and immune cell infiltration. Microbial signals may influence innate lymphoid cells (ILC3), potentially contributing to the pro-inflammatory cytokine environment in ankylosing spondylitis, although specific mechanisms require further elucidation. Mucosal plasma-cell responses, including antimicrobial antibodies, may modulate immune effector cascades; however, the pathogenic significance of anti-Klebsiella IgA in AS remains to be confirmed (26).

### 3.4 Gut Immune priming beyond translocation: molecular mimicry and context-dependent immune shifts

Some recent findings indicate that immune responses that start in the gut can be associated with not only the translocation of microbial components but also the local immune priming as well (27). There is a likelihood that a number of mechanisms leads to this process, and a number of subtypes of spondyloarthritis are affected by them. Dysbiosis may trigger mechanisms like molecular mimicry, barrier disruption and immune shifts in complex interactions, whose individual contributions vary with the context of interactions in (27, 28). This complexity highlights the importance of integrative research approaches that combine microbial, clinical, and immune profiling data to better understand disease mechanisms.

## 4 The IL-23/IL-17 axis: a key immunological pathway driving inflammation

The IL-23/IL-17 axis is a central immune pathway in spondyloarthritis and has been strongly implicated in AS-associated inflammation (7). IL-23, produced by activated macrophages and dendritic cells, promotes pathogenic Th17 differentiation and expansion.These Th17 cells, in their turn, release a great number of pro-inflammatory cytokines, which contribute to tissue damage, e.g., IL-17A, IL-17F, and IL-22.

IL-17A acts on fibroblasts, osteoblasts, and immune cells through IL-17 receptor signaling. This signaling induces chemokines, matrix-remodeling enzymes, and inflammatory mediators characteristic of SpA (29). IL-22 has context-dependent effects, including epithelial repair at barrier surfaces and possible involvement in osteoproliferative responses in AS.

Genetic studies also confirm the generalizability of the pathway, in which cytogenetic alterations in IL23R, STAT3, and TYK2 associate with increased vulnerability to AS (30). Clinical and translational studies have reported elevated IL-17/IL-23 signaling in subsets of AS patients.Th17/Treg imbalance has also been reported and may be associated with inflammatory activation and abnormal bone remodeling.

Clinically, IL-17A blockade, exemplified by secukinumab, is an established therapeutic approach for AS.Monoclonal antibodies targeting IL-17 or IL-23 pathway components have been developed and investigated in axial spondyloarthritis (31). These studies suggest that the IL-23/IL-17 axis is biologically complex and tissue-context dependent, indicating that precise therapeutic modulation may be necessary (32).

Innate-like lymphocytes, including MAIT cells, γδ T cells, and ILC3s, may contribute to IL-17 production and represent a possible link between mucosal and joint immunity.Migration of these cells from the intestinal lamina propria to peripheral sites, potentially mediated by integrins and chemokines, remains an active but incompletely proven area of research.Microbial signals may activate ILC3s and reinforce a pro-inflammatory cytokine environment, although the specific mechanisms in AS require further confirmation. Mucosal plasma-cell responses, including antimicrobial antibodies such as anti-Klebsiella IgA, may modulate immune effector cascades, but their pathogenic significance remains uncertain.

**Figure 1:**
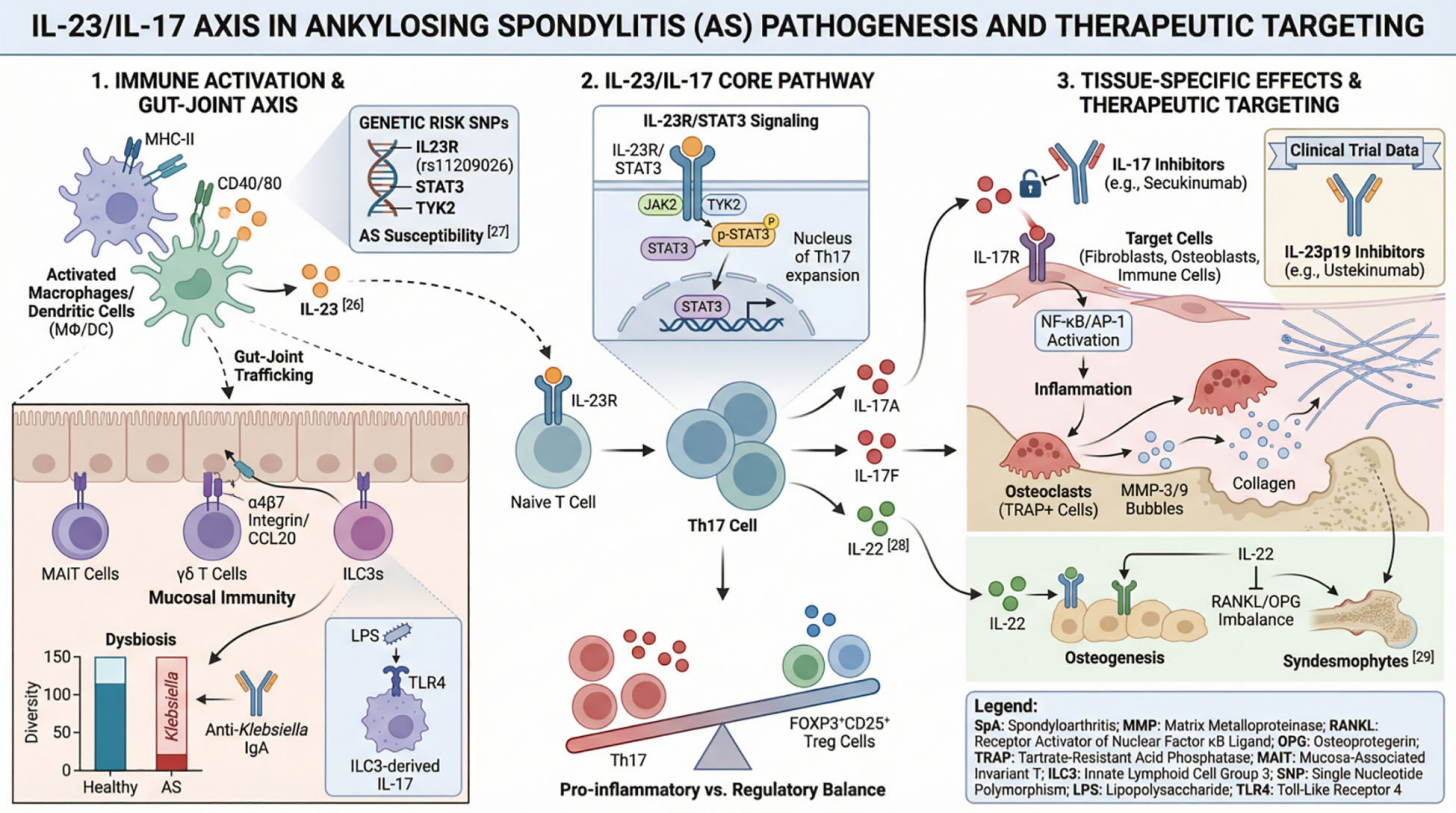
Presents a conceptual model of how the IL-23/IL-17 cascade may link gut immune activation with joint inflammation and pathological bone remodeling in AS.Intestinal microbiota dysbiosis and impaired epithelial barrier integrity are proposed to promote activation of mucosal innate-like lymphocytes, including MAIT cells, γδ T cells, and ILC3s, and may support their trafficking along the gut–joint axis. IL-23 produced by activated macrophages and dendritic cells binds IL-23R and may promote Th17 differentiation through JAK2/TYK2–STAT3 signaling, thereby influencing Th17 expansion and Th17/Treg balance. Th17 cells will then produce IL-17A, IL-17F and IL-22. The IL-17A/F IL-17R on fibroblasts, osteoblasts and immune cells stimulates NF-KB/AP-1 pathways, generates chemokines, recruits leukocytes, stimulates osteoclastogenesis, and activates matrix metalloproteinases (MMP-3/9), which leads to an amplification of joint inflammation and tissue damage. In the meantime, IL-22 is involved in repair and osteogenesis of the mucosal surface, which plays a role in the creation of the balance between RANKL/OPG and the development of syndesmophytes. The pathogenic importance of this axis is also supported by genetic predisposition loci, such as IL23R, STAT3, and TYK2 polymorphs. Clinically approved IL-17 inhibitors and investigational or context-dependent IL-23 pathway interventions target different nodes in this pathway, supporting its biological relevance while also highlighting therapeutic complexity.

Recent studies suggest that gut-derived immune responses may involve not only microbial-component translocation but also local immune priming (33). Multiple mechanisms may contribute to this process, and their relative importance may differ among spondyloarthritis subtypes. Dysbiosis in AS generally involves reduced microbial diversity and altered bacterial taxa, but these patterns vary substantially among cohorts (16). This complexity highlights the need for integrative studies combining microbial, clinical, and immune-profiling data (**Figure 1**).

## 5 HLA-B27 and the gut microbiota: coupling genetic susceptibility with environmental factors

HLA-B27 is the strongest genetic risk factor associated with AS. The arthritogenic peptide and ER-stress hypotheses are not mutually exclusive and may jointly contribute to HLA-B27-associated spondyloarthritis. The arthritogenic peptide hypothesis proposes that HLA-B27 contributes to spondyloarthritis by presenting disease-relevant peptides to T cells (34). In contrast, the ER-stress hypothesis emphasizes HLA-B27 misfolding and downstream inflammatory signaling, including possible activation of the IL-23/IL-17 axis during disease progression (35).

Gut microbiota and their metabolites may influence AS pathobiology through mucosal immune activation, increased intestinal permeability, and interactions with host genetic susceptibility (8). These interactions provide a framework linking microbial composition with epithelial barrier integrity and immune activation in AS (8). However, the relative contribution of each component likely varies across patients, disease stages, and treatment contexts (8).

Collectively, these findings support a bidirectional model in which HLA-B27-related host immunity may shape microbial composition. Conversely, microbial factors may modulate immune pathways associated with HLA-B27 (36). This complex interplay of genetics and microbiota is of interest in the pathobiology of AS unraveling (8).

In addition, innate-like lymphocytes including the MAIT cells, 7delta T cells and ILC3 cells also play a role in the production of IL-17 (37). The potential migration of intestinal intraepithelial lymphocytes or other cytokine-competent lymphocytes to joint tissues is an emerging area of investigation with possible therapeutic implications (38).

## 6 Microbial metabolites: functional hubs linking the microbiota and immunity

Beyond microbial composition, SCFAs and tryptophan derivatives may function as immunometabolic mediators linking gut microbiota with mucosal and systemic immune regulation (39). SCFAs, including butyrate, propionate, and acetate, contribute to epithelial barrier integrity, Treg differentiation, and suppression of pro-inflammatory cytokine production through G-protein-coupled receptors and histone deacetylase inhibition (40, 41).

Butyrate may influence immune-cell fate and function through epigenetic regulation, as shown in inflammatory disease models, although direct evidence in AS remains limited (42). Although propionate and acetate play slightly different roles, they also play a role in the control of immunity (40, 41). Both experiments in animal models and ex vivo of the human system have shown that supplementing with either SCFAs or SCFA-generating bacteria can inhibit Th17 polarization and reduce production of inflammatory cytokines (43).

Tryptophan metabolism is also relevant to mucosal immune regulation. Microbiota-derived indole metabolites can activate aryl hydrocarbon receptor (AhR) signaling, thereby promoting IL-22 production and epithelial repair (44, 45). Although IL-22 supports tissue repair, it may also participate in osteoproliferative processes in AS, indicating context-dependent effects. Besides, AhR signaling has an influence on regulatory immune pathways, in particular, stimulating IL-10 and indoleamine 2, 3-dioxygenase (IDO) (46).

The metabolomic profiling of the AS patients has demonstrated disturbed indole signatures especially in the indole-3-acetate (IAA) and indole-3-aldehyde (I3Ald) lineages of tryptophan metabolites, which highlights the importance of the microbial metabolites in regulating the immune system (47). Although these findings are encouraging, additional functional studies are needed to determine causal relationships and therapeutic potential (48).

**Figure 2:**
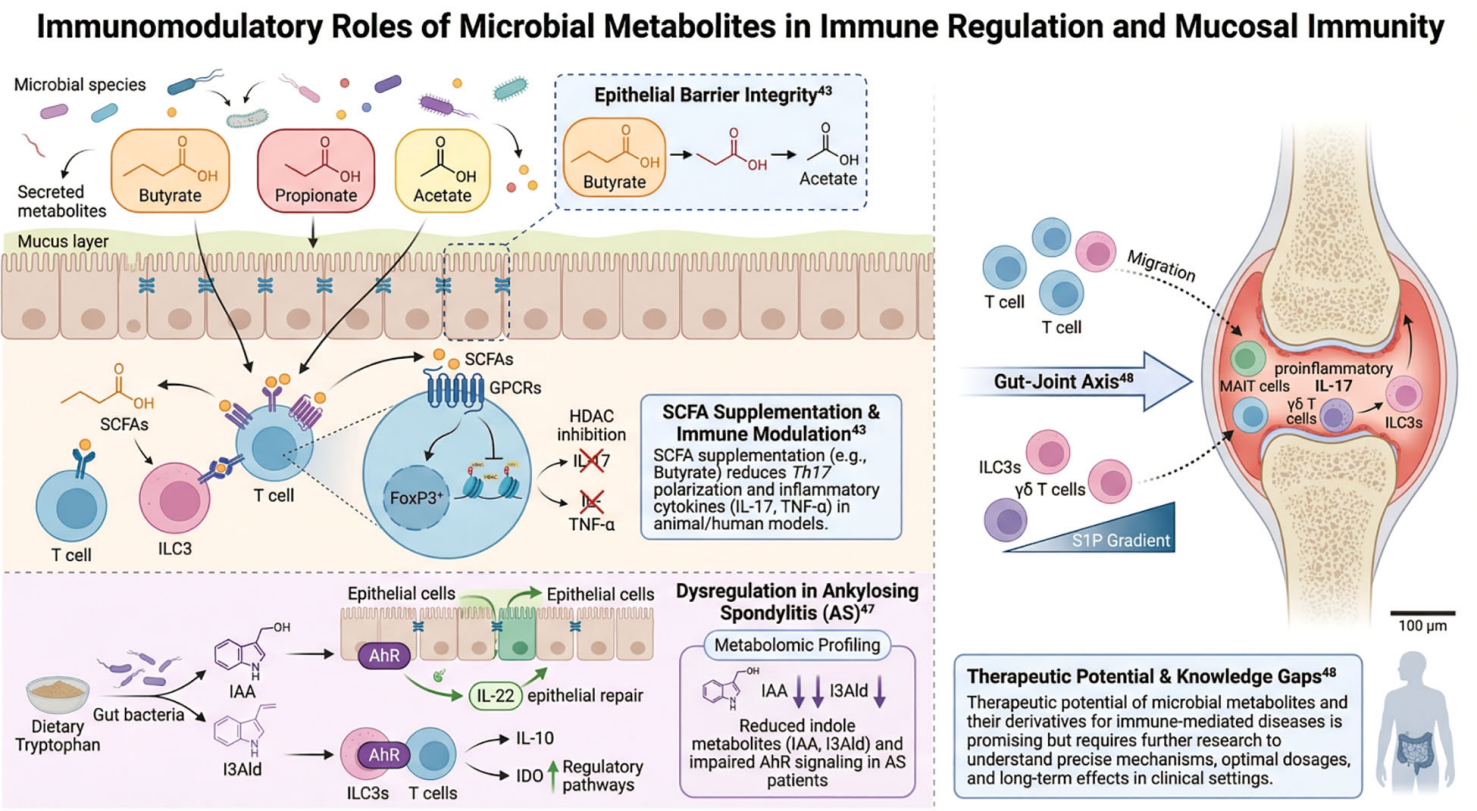
Summarizes a proposed mechanism by which microbial metabolites may influence epithelial barrier integrity, mucosal immunity, and systemic immune responses relevant to AS.Commensal bacteria produce short-chain fatty acids (SCFA) such as butyrate, propionate, and acetate, which improve the integrity of epithelium and regulate intestinal immune cells via the G protein-linked receptors (GPCRs). SCFAs can inhibit histone deacetylase (HDAC) activity, promote FoxP3+ regulatory T-cell differentiation, and reduce production of pro-inflammatory cytokines such as IL-17 and TNF-α, thereby potentially limiting Th17-mediated inflammation.Experimental and limited clinical evidence suggests that SCFA supplementation or SCFA-producing bacteria may reduce Th17 polarization and inflammatory responses, although AS-specific clinical evidence remains insufficient.

Innate-like lymphocytes, including MAIT cells, γδ T cells, and ILC3s, may participate in IL-17 production and gut–joint immune trafficking (49, 50). The potential trafficking of these cells from the intestinal lamina propria to peripheral sites, mediated by S1P, integrins, and chemokines, remains a promising but still developing research area (51) (**Figure 2**).

Simultaneously, tryptophan is a dietary amino acid metabolized by gut bacteria into tryptophan indole derivatives, such as indole-3-acetic acid (IAA) and indole-3-aldehyde (I3Ald), which activate the aryl hydrocarbon receptor (AhR) in epithelial cells and innate lymphoid cells (ILC3s). AhR signaling promotes IL-22 production, epithelial repair, and regulatory mediators such as IL-10 and IDO, thereby supporting mucosal immune homeostasis.

In AS, metabolomic profiling suggests reduced indole metabolites and altered AhR-related signaling, which may contribute to epithelial dysfunction and immune imbalance.Altered metabolite-mediated immune responses may support activation and possible gut–joint trafficking of T cells, MAIT cells, γδ T cells, and ILC3s within a pro-inflammatory cytokine environment, particularly one enriched in IL-17.Overall, the figure highlights the potential role of microbial metabolites in mucosal and systemic immune homeostasis, while emphasizing remaining gaps regarding specific mechanisms, optimal interventions, and long-term clinical efficacy.

## 7 An integrative framework: therapeutic strategies and translational prospects

Preclinical and clinical studies suggest that gut–immune interactions may contribute to AS pathobiology, but the strength of evidence varies across mechanisms and study types (42). The microbial communities associated with AS have been implicated in the immune pathogenesis involving the IL-23/IL-17 pathway and genetic factors such as HLA-B27 and ERAP1 (52). Moreover, studies have identified alterations in the gut microbiota composition and genetic and epigenetic factors involved in AS pathogenesis (17, 53). These findings support the hypothesis that gut dysregulation may contribute to systemic inflammation; however, they do not establish gut dysbiosis as the primary initiating factor (54).

Therapeutic evidence helps distinguish established immune-targeted treatments from experimental microbiota-directed approaches. Anti-TNF and IL-17 blocking therapies have been associated with complex effects on intestinal inflammation and microbiota composition, including paradoxical reactions in some patients (55, 56). In contrast, probiotics, prebiotics, and fecal microbiota transplantation remain experimental in AS, with most clinical evidence derived from gastrointestinal or non-axial inflammatory conditions.

Future therapeutic strategies may involve combined modulation of immune and microbial pathways, but such approaches require prospective clinical validation. Potential approaches include dietary strategies to enhance SCFA production, cytokine-pathway modulation with JAK inhibitors, and blockade of leukocyte trafficking through integrin-directed mechanisms (39, 57). These approaches are promising but should currently be viewed as adjunctive or experimental rather than established AS treatments (58).

A key unresolved question is whether innate-like lymphocytes migrate from the intestinal lamina propria to peripheral tissues in AS and whether this process is controlled by integrins and chemokines. AS-associated microbial profiles often show reduced diversity and inflammatory features, but these associations require validation in longitudinal cohorts (16). SCFAs derived from the gut microbiota promote IL-22 production by CD4+ T cells and innate lymphoid cells, contributing to intestinal homeostasis and protection against inflammation (39).

Mucosal immunity is also influenced by tryptophan metabolism, whose derivatives can activate AhR signaling and contribute to gastrointestinal homeostasis (59). Although IL-22 is important for tissue repair, its potential contribution to osteoproliferation in AS warrants further investigation. Gut microbiota-derived tryptophan metabolites may regulate AhR signaling and thereby affect gastrointestinal homeostasis (59).

Metagenomic studies in AS have identified changes in microbial composition and functional pathways, supporting a role for microbial factors in immune modulation (17). Although these findings are promising, further functional and longitudinal studies are required to clarify causal relationships and therapeutic value (48).

**Figure 3:**
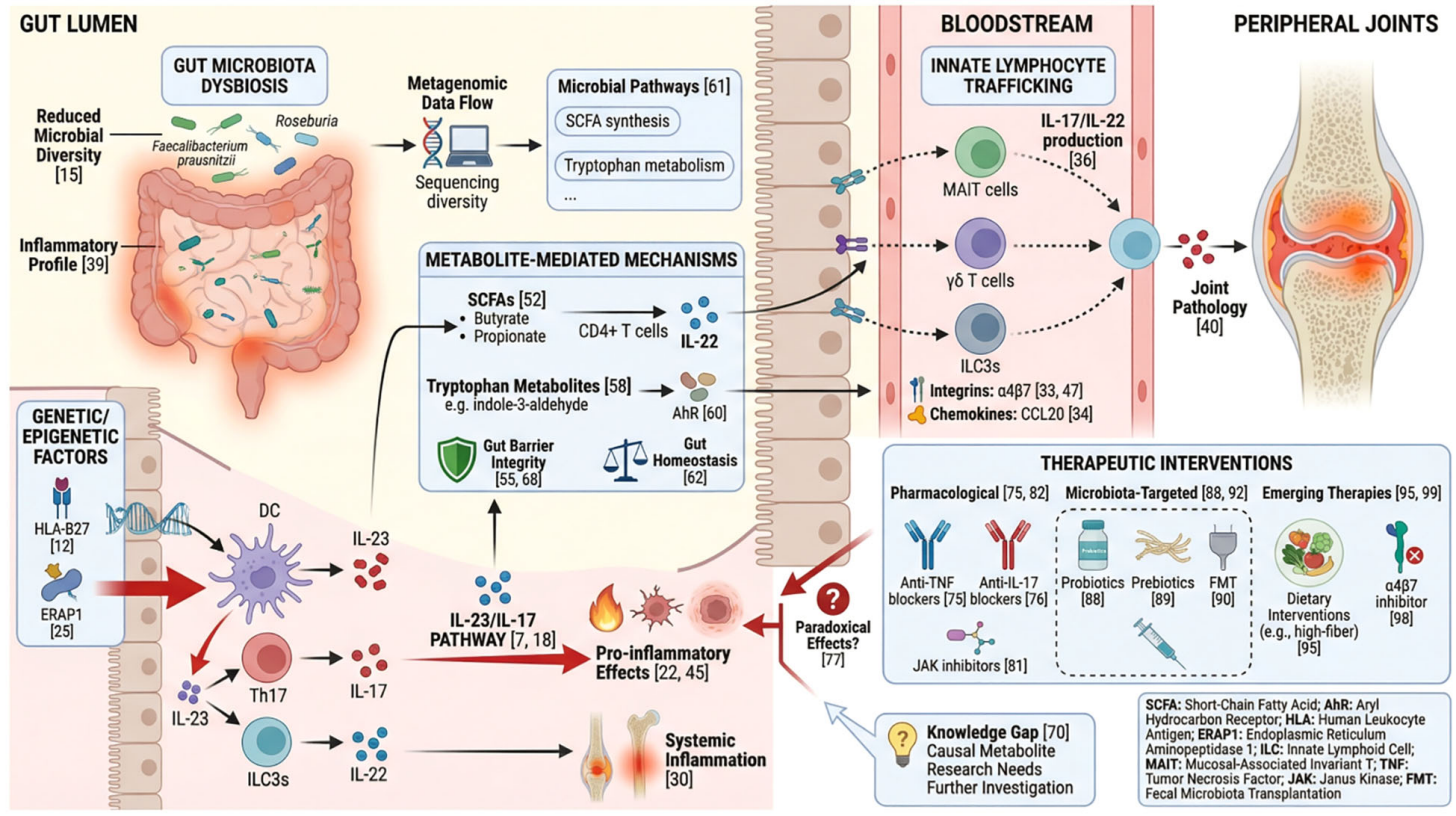
Provides a conceptual summary of the proposed gut–joint axis in AS, linking gut microbiota dysbiosis, metabolite alterations, mucosal immune dysregulation, and joint inflammation. Furthermore, the decreased diversity of microbial communities and a proinflammatory state of the gut lumen are linked to the altered microbial functional pathways, identified through metagenomic sequencing (e.g. alterations in short-chain fatty acid [SCFA] production and tryptophan metabolism). These metabolic disturbances may affect epithelial barrier function and gut homeostasis. SCFAs, especially butyrate and propionate, support epithelial barrier integrity and may induce regulatory immune programs, whereas dysregulated tryptophan-derived metabolites, such as indole-3-aldehyde, may signal through AhR to regulate IL-22 secretion and epithelial repair.

MAIT cells, γδ T cells, and ILC3s can produce IL-17 and may participate in gut–joint immune trafficking (60). The migration of intestinal lamina propria immune cells to peripheral sites through integrin- and chemokine-mediated pathways remains an active area of research (**Figure 3**).

Host genetic and epigenetic susceptibility factors, including HLA-B27 and ERAP1, may interact with mucosal immune activation and promote IL-23 production by dendritic cells.IL-23 can promote Th17 and ILC3 responses, leading to IL-17 and IL-22 production and downstream inflammatory effects that may extend from the gut to systemic sites.At the same time, innate-like lymphocytes, including MAIT cells, γδ T cells, and ILC3s, may be activated in the intestinal compartment and potentially traffic through the bloodstream to peripheral joints under the influence of integrins and chemokine signals.These cells may contribute to local IL-17/IL-22 production in joint tissues and thereby promote inflammation, but this mechanism should be interpreted as a proposed model rather than a proven pathway.

Finally, the figure distinguishes established immune-targeted interventions, such as anti-TNF, anti-IL-17, and JAK inhibitors, from microbiota-directed strategies, including probiotics, prebiotics, FMT, and dietary interventions, which remain experimental in AS.The diagram also highlights unresolved issues, including paradoxical therapeutic effects, uncertain causal metabolite pathways, and the need for mechanistic and translational studies.

## 8 Conclusion

Recent studies have highlighted associations among gut microbiota, epithelial barrier integrity, mucosal immunity, and AS (53, 61). The gut–joint axis can be viewed as a dynamic, bidirectional framework in which dysbiosis, barrier dysfunction, and immune activation may interact to shape disease pathobiology (62, 63). Further clarification of these interactions may improve understanding of AS mechanisms and support the development of more targeted therapeutic strategies (4).

Future research should prioritize longitudinal, multi-omic studies in well-defined patient cohorts to identify early biomarkers, disease endotypes, and mechanistic targets. Such studies will be essential for translating microbiome research into clinically applicable interventions for AS and other immune-mediated diseases (64).
